# SOX9‐activated PXN‐AS1 promotes the tumorigenesis of glioblastoma by EZH2‐mediated methylation of DKK1

**DOI:** 10.1111/jcmm.15189

**Published:** 2020-04-23

**Authors:** Hongjin Chen, Guoqiang Hou, Jian Yang, Weilin Chen, Liemei Guo, Qin Mao, Jianwei Ge, Xiaohua Zhang

**Affiliations:** ^1^ Department of Neurosurgery School of Medicine Renji Hospital Jiaotong University Shanghai China; ^2^ Department of Pediatric Neurosurgery Xin Hua Hospital affiliated to School of Medicine Shanghai Jiao Tong University Shanghai China

**Keywords:** DKK1, EZH2, glioblastoma, PXN‐AS1, WNT pathway

## Abstract

Increasing evidence has validated the essential regulation of long non‐coding RNAs (lncRNAs) in the biological process of tumours. LncRNA PXN‐AS1 has been discovered to be as a tumour suppressor in pancreatic cancer; however, its function and mechanism remain greatly unknown in glioblastoma (GBM). Our present study indicated that PXN‐AS1 was highly expressed in GBM tissues and cells. Besides, the knock‐down of PXN‐AS1 was closely associated with the inhibitory proliferation and inducing apoptosis of GBM cells. PXN‐AS1 inhibition was also found to restrain GBM tumour growth. Importantly, SOX9 functioned as a transcription factor and activated PXN‐AS1 expression, and overexpressed PXN‐AS1 rescued the inhibitory role of down‐regulated SOX9 in GBM cell growth. Subsequently, it was discovered that PXN‐AS1 activated Wnt/β‐catenin pathway. DKK1 was widely known as an inhibitor gene of Wnt/β‐catenin pathway, and its expression was negatively associated with PXN‐AS1 and SOX9. Interestingly, we found that PXN‐AS1 could recruit EZH2 to mediate the H3K27me3 level of DKK1 promoter. Restoration experiments manifested that DKK1 knock‐down counteracted PXN‐AS1 depletion‐mediated repression in GBM cell growth. All facts pointed out that PXN‐AS1 might be of importance in exploring the therapeutic strategies of GBM.

## 1 INTRODUCTION

Glioblastoma (GBM) is an aggressive tumour derived from central nervous system and characterized by high recurrence rate.^1^ The generation of new blood vessels is one of the most significant characteristics of GBM. In the meantime, as a normal angiogenic solid tumour, the growth and development of GBM are correlated with the nourishment of blood vessels. In recent years, great advances have been made in surgery, radiotherapy and chemotherapy; therefore, the treatment of GBM was improved to a certain degree. However, the long‐term survival rate remains unsatisfied due to the invasive property of GBM.^2^ Existing studies have revealed that GBM progression is correlated with the regulatory networks of genes.^3^, ^4^ Hence, it is of great significance to investigate novel biomarkers underlying GBM tumorigenesis and explore the potential mechanism which might be useful in improving therapeutic strategies.

Long non‐coding RNAs (lncRNAs) are one type of transcripts whose length are more than 200 nucleotides and have restriction in the ability of protein‐coding.^5^ Owing to their modulation in biological behaviours, increasing lncRNAs have attracted people's attention.^6^ Extended evidence has confirmed that lncRNAs are closely associated with the progression of human diseases, particularly with cancers, through multiple mechanisms, including chromatin modification, transcriptional and post‐transcriptional processing, and interacting with specific proteins.^7^, ^8^, ^9^, ^10^ Over the past few years, it has been validated that lncRNAs could be diagnostic or prognostic biomarkers in diverse cancers. For example, lncRNA LINC00978 functioned as a diagnostic biomarker and promotes cancer growth in gastric cancer.^11^ LncRNA FOXP4‐AS1 acts as a prognostic factor in colorectal cancer and modulates colorectal cancer cell proliferation and apoptosis.^12^ A wide range of lncRNAs have been reported in GBM. For example, LINC00470 mediated GBM cell autophagy by activating AKT.^13^ LncRNA HOTAIRM1 was overexpressed in GBM and promotes cell invasion and tumour growth via up‐regulating HOXA1.^14^ LncRNA PXN antisense RNA 1 (PXN‐AS1) was reported to be down‐regulated in pancreatic cancer cells and suppressed cancer progression.^15^ However, the expression of PXN‐AS1 was found to be up‐regulated in GBM tissues through GEPIA database. Therefore, we wondered whether PXN‐AS1 exerted reverse functions in GBM.

In this study, we explored the biological function and molecular mechanism of PXN‐AS1 in GBM. It was found that PXN‐AS1 was up‐regulated in GBM cells and silenced PXN‐AS1 resulted in the repressive cell proliferation and induced cell apoptosis. Mechanistically, SOX9‐activated PXN‐AS1 promoted the GBM progression by epigenetically silencing DKK1 and thereby activated Wnt/β‐catenin pathway.

## 2 MATERIALS AND METHODS

### 2.1 Cell lines and reagent

Four human GBM cells (A172, U251, U87, LN229) and normal human astrocyte cell (NHA), all from ATCC (Rockville, Maryland), were cultivated in the DMEM (Invitrogen) with 95% air and 5% CO_2_ at 37°C. 10% FBS (HyClone) and 1% Pen/Strep solution (Invitrogen) served as the supplements for DMEM. Wnt/β‐catenin pathway activator (LiCl; 30 μmol/L) was commercially acquired from Sigma‐Aldrich to treat U251 and U87 cells.

### 2.2 Quantitative real‐time PCR (qRT‐PCR)

Total RNA was extracted from U251 and U87 cells following the manual of RNeasy Mini Kit (Qiagen) for synthesizing cDNA templates using Reverse Transcription Kit (Toyobo). Gene expression was quantified by Bio‐Rad SYBR Green Super Mix (Bio‐Rad) and calculated by 2^−ΔΔCT^ method, relative to GAPDH or U6. The primers used here were shown in Table S1.

### 2.3 Cell transfection

The shRNAs specific to PXN‐AS1, SOX9, EZH2 and DKK1 were procured from Genepharma for gene silencing, as well as non‐specific shRNAs as negative control (NC). The pcDNA3.1/SOX9, pcDNA3.1/PXN‐AS1, pcDNA3.1/DKK1 and empty pcDNA3.1 vectors were produced by Genechem. Lipofectamine 2000 (Invitrogen) was use for the 48 hours of transfection.

### 2.4 Colony formation

The transfected U251 and U87 cells were plated at 500 cells/well in the 6‐well plates. Following 14 days of incubation, clones were treated with 0.5% crystal violet in 4% paraformaldehyde.

### 2.5 5‐Ethynyl‐2ʹ‐deoxyuridine (EdU)

EdU Apollo in‐vitro flow cytometry kit (RiboBio) was used to verify cell proliferation of U251 and U87 cells. Treated about 48 hours, EdU solution (50 µmol/L) was added and cells were incubated for 2 hours. Later, U251 and U87 cells were washed in PBS and fixed by 4% paraformaldehyde. Subsequently, Apollo was utilized for staining cells. Before cell nucleus was observed, the stained cells were processed by Hoechst for half an hour. Then, cell proliferation was observed using a high‐power microscope and photographed on MetaXpress software (Molecular Devices).

### 2.6 Immunofluorescence (IF) staining

GBM cells on coverslips were treated with 4% paraformaldehyde, then with 0.1% Triton X‐100 and 0.5% BSA (Amresco). Samples were probed with primary antibody against Ki‐67 (ab92742, Abcam) for 1 hours, followed by incubation with HRP‐conjugated secondary antibody (ab6789, Abcam) after washing in PBS. Stained cells were observed by fluorescence microscope.

### 2.7 JC‐1 assay

Recently, JC‐1 assay has been usually carried out to examine early apoptosis event in GBM cells under different conditions since this experiment could detect changes in mitochondrial membrane potential, which is a hallmark of early cell apoptosis.^16^ When the mitochondrial membrane potential is high (namely apoptosis rate is low), JC‐1 accumulates in the matrix of mitochondria, thus producing red fluorescence. On the contrary, JC‐1 cannot concentrate in the matrix of mitochondria when the mitochondrial membrane potential is low (cells with high apoptosis rates), finally resulting in green fluorescence. In this study, JC‐1 assay was implemented in GBM cells in line with the instruction of JC‐1 detection kit (Beyotime). Following fluorescence labelling, samples were rinsed in PBS for analysis with EnSpire Reader (PerkinElmer). The JC‐1 ratio was evaluated as the rate of red signals relative to green signals.

### 2.8 Western blot

The protein extracts from GBM cells were loaded to 12% SDS‐PAGE, shifted onto PVDF membranes (Millipore) and then treated with 5% BSA. The primary antibodies (dilution 1:2000) against loading control GAPDH (ab125247, Abcam), Bax (ab32503, Abcam), Bcl‐2 (ab32124), β‐catenin (ab6302, Abcam), cyclin D1 (ab40754, Abcam), c‐myc (ab32072, Abcam), DKK1 (ab109416, Abcam) and corresponding secondary antibodies (ab6789, Abcam) were all procured from Abcam. Band density was finally analysed by ECL detection system (Thermo Fisher Scientific).

### 2.9 Flow cytometer of apoptosis

After transfection, cells were first fixed for 1 hours on ice and then treated with Annexin V‐FITC/PI (BD Biosciences, Erembodegem, Belgium) for 15 minutes in the dark. Cell apoptosis rate was monitored by flow cytometry (BD Biosciences).

### 2.10 Animal study

10 six‐week‐old male BALB/c nude mice were procured from Shanghai SIPPR‐BK Laboratory Animal (Shanghai, China) and housed under SPF condition, with the approval from the Use Committee for Animal Care of Renji Hospital. Each mouse was subcutaneously inoculated with 5 × 10^6^ GBM cells. Tumour volume was monitored every 4 days according to the calculating formula: Volume = 0.5 × length × width^2^. Tumour samples were excised from mice 28 days later for weight assessment.

### 2.11 Immunohistochemistry

The tumour tissue samples from animal study were fixed with 4% paraformaldehyde and then embedded in paraffin for cutting. Consecutive 4‐μm thick sections were acquired for IHC analysis using anti‐Ki‐67 antibody (Santa Cruz Biotechnology).

### 2.12 In situ hybridization assay

The fresh tumour tissue samples were cultured in 0.5% Triton X‐100 and PBS for incubation with the PXN‐AS1 FISH probe (RiboBio) in hybridization solution. After DAPI staining, samples were analysed with fluorescence microscope.

### 2.13 Chromatin immunoprecipitation

After cross‐linking, the chromatin samples were prepared for sonication to 200‐1000‐bp fragments and then immunoprecipitated with the 2 μg of specific antibodies (Millipore) and 30 μL of magnetic beads. qRT‐PCR was followed finally.

### 2.14 Dual‐luciferase reporter gene assay

The wild‐type (WT) or mutated (Mut) SOX9 binding sites to PXN‐AS1 promoter were severally inserted into luciferase reporter pGL3 (Promega), termed promoter‐WT/Mut. U251 and U87 cells in the 24‐well plates were used for cotransfection with promoter‐WT/Mut reporter vectors, pRL‐TK‐Renilla plasmid (Promega) and SOX9 silencing plasmids or SOX9 overexpression plasmids. Besides, the Wnt/β‐catenin signalling reporters TOP Flash/FOP Flash (Promega) were acquired and transfected into GBM cells with sh‐PXN‐AS1 and sh‐NC or sh‐SOX9 or sh‐NC. Dual‐Luciferase Reporter Assay System (Promega) was applied after 48 hours of transfection.

### 2.15 Subcellular fractionation

The GBM cells in pre‐chilled PBS were centrifuged for incubation in cell fractionation buffer for cell cytoplasm and cell disruption buffer for cell nuclei. Isolated RNAs were finally detected by qRT‐PCR.

### 2.16 RNA immunoprecipitation

Magna RNA‐binding protein immunoprecipitation kit (Millipore) was employed to perform RNA immunoprecipitation (RIP) assays. In brief, the cultured GBM cells were first collected from RIP lysis buffer and then incubated in RIP buffer adding the beads bound the antibodies against human Ago2 (ab32381, Abcam) and normal rabbit IgG (ab172730, Abcam) for 2 hours. The final precipitated RNAs were subjected to qRT‐PCR.

### 2.17 Statistical analyses

Results were expressed as the mean ± standard deviation (SD) of bio‐triple repeats. All data differences in this study were analysed through Student's *t* test or one‐way analysis of variance using GraphPad Prism 6 software (GraphPad Software, Inc). The statistical significance was specified as the value of *P* < .05. Each experiment was repeated three times.

## 3 RESULTS

### 3.1 PXN‐AS1 is overexpressed in GBM cells and enhances cell proliferation and restrains cell apoptosis

**FIGURE 1:**
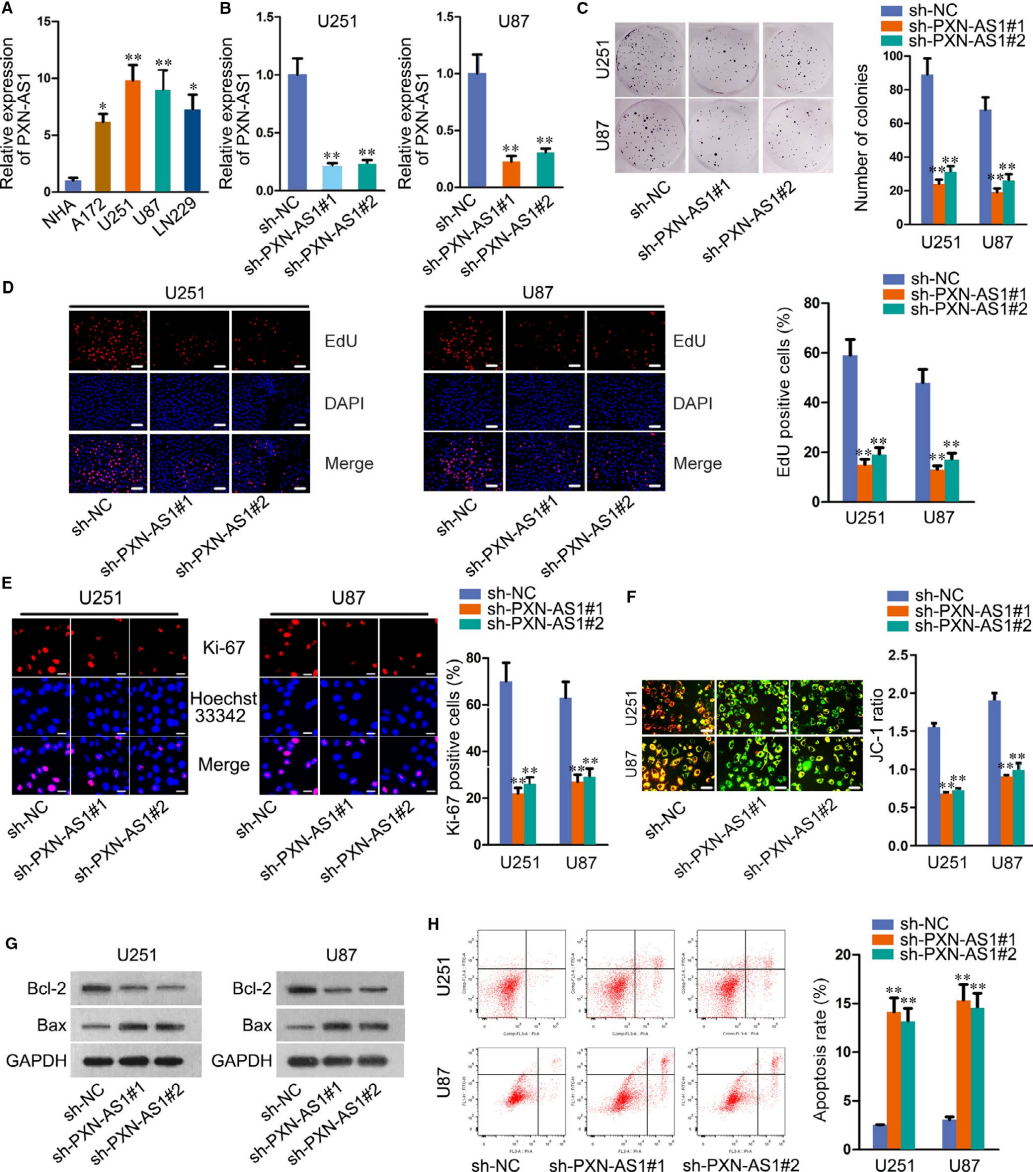
PXN‐AS1 is overexpressed in GBM cells and enhances cell proliferation and restrains cell apoptosis. A, PXN‐AS1 relative expression in human GBM cell lines (A172, U251, U87 and LN229) and normal human astrocyte cell line NHA. B, PXN‐AS1 expression in GBM cells transfected with sh‐PXN‐AS1 (sh‐PXN‐AS1#1, sh‐PXN‐AS1#2). C‐E, The proliferative ability of PXN‐AS1 silenced GBM cells was measured by performing colony formation assay, EdU assay and immunofluorescence. Scale bar = 100 μm. F‐H, JC‐1, Western blot and flow cytometry assays were conducted to evaluate cell apoptosis upon PXN‐AS1 knock‐down. Scale bar = 100 μm. *P < .05, **P < .01

Through GEPIA (http://gepia.cancer‐pku.cn/), we found that PXN‐AS1 was up‐regulated in GBM tissues compared to the paired normal tissues (Figure S1A). To verify this, we detected PXN‐AS1 expression in GBM cells (A172, U251, U87, LN229), and normal human astrocyte cell (NHA) was taken as a reference. The results manifested notable overexpression of PXN‐AS1 in GBM cells, especially in U251 and U87 cells (Figure 1A). Therefore, we selected U251 and U87 cells for further experiments. To explore the role of PXN‐AS1 in GBM progression, we reduced PXN‐AS1 expression in U251 and U87 cells by transfecting two specific PXN‐AS1 shRNAs (sh‐PXN‐AS1#1, sh‐PXN‐AS1#2). The results showed that PXN‐AS1 expression was remarkably reduced in sh‐PXN‐AS1#1/2 transfected cells (Figure 1B). Subsequently, loss‐of‐function assays were designed and carried out. Colony formation, EdU and immunofluorescence assays were performed to test the effect of PXN‐AS1 depletion on cell proliferation. As a result, the proliferative capacity of U251 and U87 cells was considerably weakened upon PXN‐AS1 knock‐down (Figure 1C‐E). JC‐1 assay data indicated that the knock‐down of PXN‐AS1 induced cell apoptosis in U251 and U87 cells (Figure 1F). Through Western blot assay, we observed decreased Bcl‐2 level and increased Bax level in sh‐PXN‐AS1#1/2‐transfected cells (Figure 1G). Flow cytometry analysis further confirmed the inhibitory role of silenced PXN‐AS1 in cell apoptosis (Figure 1H). All data indicated that PXN‐AS1 was overexpressed in GBM cells and enhanced cell proliferation and restrained cell apoptosis.

### 3.2 PXN‐AS1 facilitates tumour growth in GBM

**FIGURE 2:**
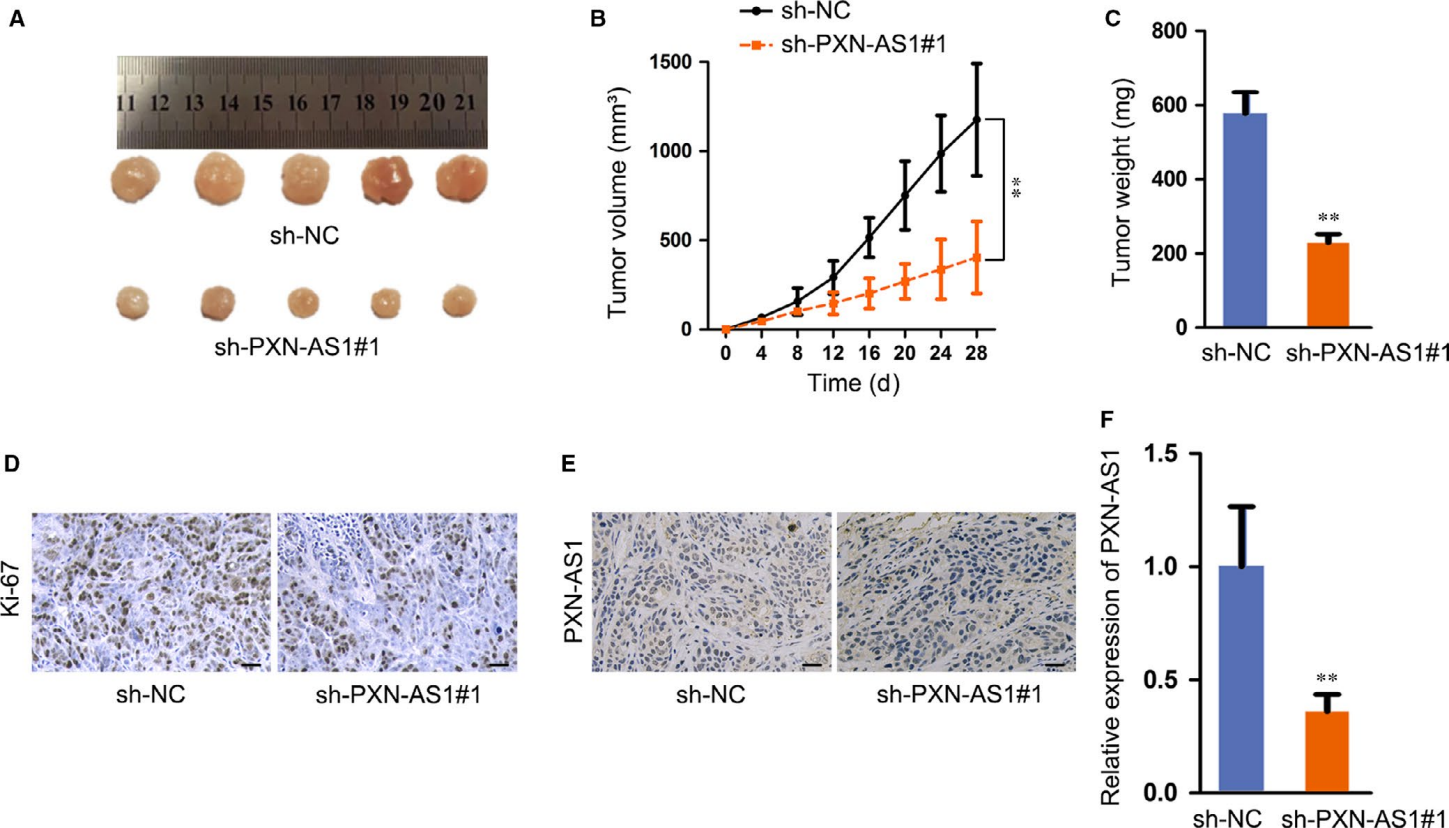
PXN‐AS1 facilitates tumour growth in GBM. A, U251 cells transfected with sh‐PXN‐AS1 and sh‐NC were subcutaneously injected into nude mice, and tumours were removed after 28 d. B‐C, Tumour volume and tumour weight were analysed between sh‐PXN‐AS1 and sh‐NC groups. D, Immunohistochemical staining of Ki‐67 in tumours. Scale bar = 100 μm. E, In situ hybridization was performed to detect PXN‐AS1 expression in the nude mice. Scale bar = 100μm. F, PXN‐AS1 expression in vivo was indicated by qRT‐PCR analysis. **P < .01

Next, we noticed the function of PXN‐AS1 on GBM tumour growth in vivo, and U251 cells transfected with sh‐PXN‐AS1 or sh‐NC were subcutaneously injected into nude mice. Observed in 28 days, the tumours were removed, and the weight was measured. As expected, the tumour growth rate was slower, and the final volume and weight in sh‐PXN‐AS1 group were lower than those in sh‐NC group (Figure 2A‐C). The results of immunohistochemistry (IHC) assay depicted that the tumours developed from sh‐PXN‐AS1 cells demonstrated reduced Ki‐67 staining in comparison with the tumours from sh‐NC cells (Figure 2D). Through In situ hybridization (ISH) assay, PXN‐AS1 expression was diminished in sh‐PXN‐AS1 group compared to NC group (Figure 2E). In addition, qRT‐PCR analysis implied that PXN‐AS1 expression level showed significant decrease upon PXN‐AS1 knock‐down in vivo (Figure 2F). Taken together, PXN‐AS1 facilitates tumour growth in GBM.

### 3.3 SOX9 interacts with PXN‐AS1 promoter

**FIGURE 3:**
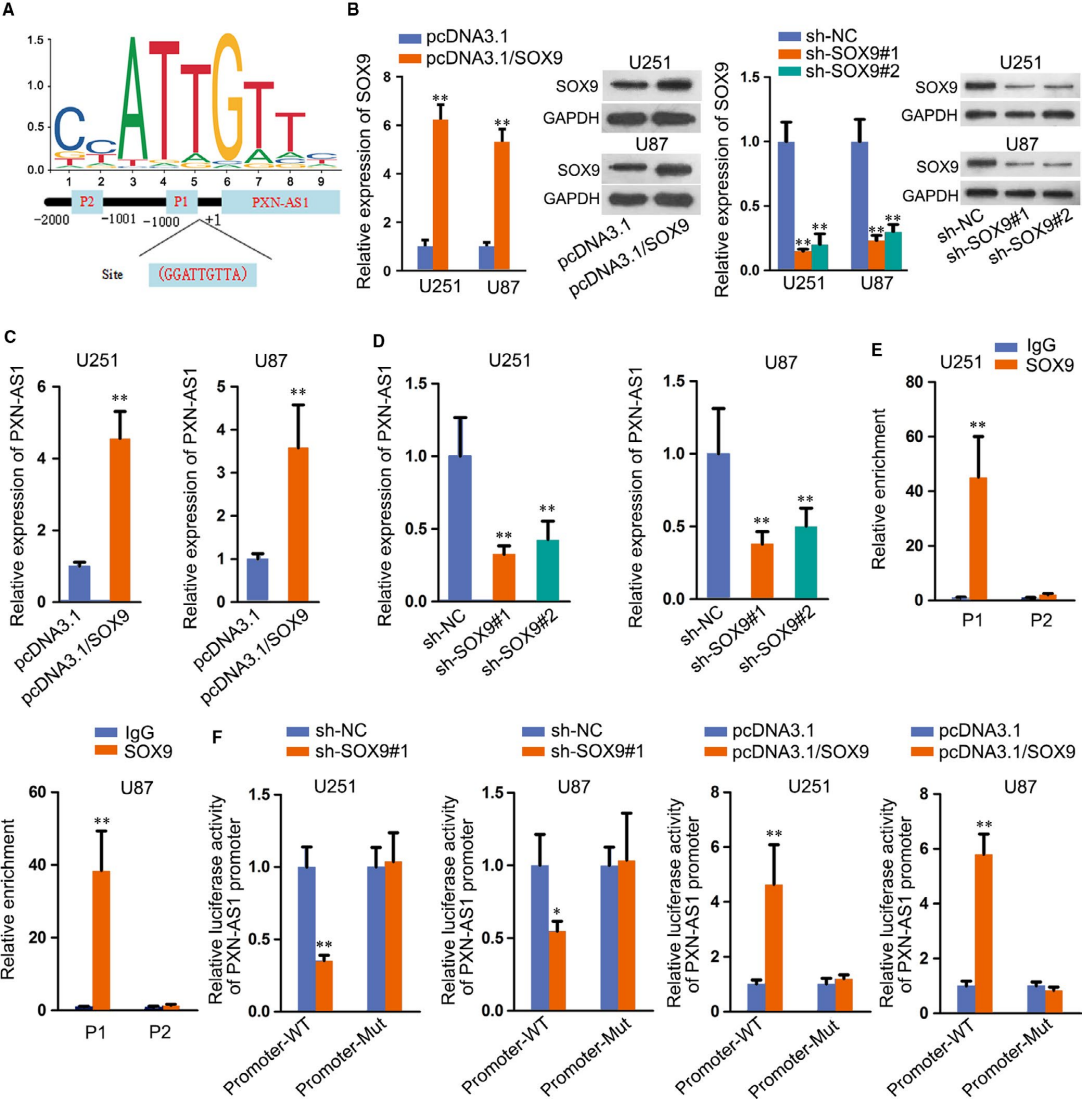
SOX9 interacts with PXN‐AS1 promoter. A, SOX9 DNA motif and binding site in PXN‐AS1 promoter. B, SOX9 expression was tested by qRT‐PCR and Western blot in cells separately transfected with pcDNA3.1/SOX9 and sh‐SOX9#1/2. C, qRT‐PCR showed the effect of SOX9 overexpression on PXN‐AS1 expression. D, PXN‐AS1 expression was detected via qRT‐PCR in SOX9 down‐regulated cells. E, ChIP assay was carried out to examine the interaction between SOX9 and PXN‐AS1 promoter. F, SOX9 was further checked to combine with PXN‐AS1 promoter by luciferase reporter assay. *P < .05, **P < .01

Subsequently, we investigated the mechanism that correlated with the up‐regulation of PXN‐AS1. Extensive reports have suggested that transcription factors could result in the abnormal expressions of lncRNAs in human cancers. SOX9 functioned as a transcription factor and widely reported in GBM^17^, ^18^; hence, we wondered whether SOX9 transcriptionally activated PXN‐AS1 expression in GBM. According to JASPAR database (http://jaspar.genereg.net/), two potential sites were predicted for the binding of SOX9 to PXN‐AS1 promoter (Figure 3A). Then, overexpression and knock‐down efficiency of SOX9 was detected by both qRT‐PCR and Western blot, and SOX9 expression was obviously up‐regulated by pcDNA3.1/SOX9 and reduced after transfecting sh‐SOX9 (Figure 3B). Results of qRT‐PCR demonstrated that PXN‐AS1 expression was elevated by SOX9 overexpression and reduced by SOX9 knock‐down (Figure 3C‐D). Chromatin immunoprecipitation (ChIP) assay elucidated that SOX9 bound to P1 of PXN‐AS1 promoter region (Figure 3E). Results of luciferase reporter assay further validated that luciferase activity of promoter‐WT was inhibited in sh‐SOX9 transfected cells and augmented in cells transfected with pcDNA3.1/SOX9 (Figure 3F). Also, we discovered the expression of SOX9 in in vivo tumours was not affected by PXN‐AS1 inhibition (Figure S1B), suggesting SOX9 was the upstream of PXN‐AS1 in GBM. To further make sure whether PXN‐AS1 was the downstream of SOX9 in GBM, we overexpressed PXN‐AS1 expression in GBM cells to conduct the following rescue assays (Figure S2A). As observed in Figure S2B‐D, the suppressed cell proliferation caused by silenced SOX9 was recovered by up‐regulated PXN‐AS1. In addition, PXN‐AS1 overexpression also restored the boosted role of SOX9 knock‐down in cell apoptosis (Figure S2E‐G). In brief, SOX9 interacts with PXN‐AS1 promoter and activates the expression of PXN‐AS1.

### 3.4 PXN‐AS1 activates WNT signalling pathway

**FIGURE 4:**
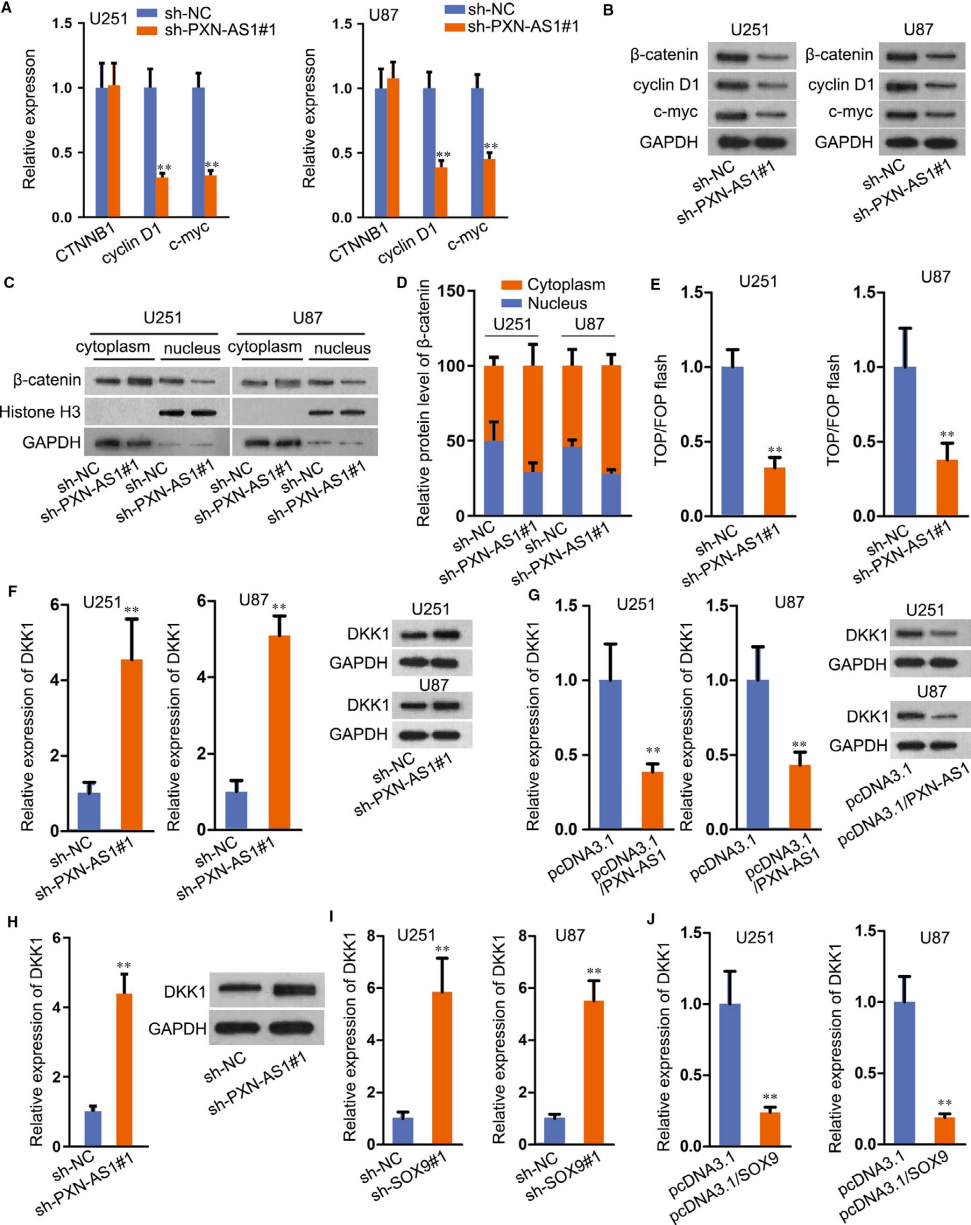
PXN‐AS1 activates Wnt/β‐catenin pathway. A‐B, The related genes mRNA and protein levels of Wnt/β‐catenin pathway were measured by qRT‐PCR and Western blot assay. C‐D, Western blot after subcellular fractionation assay was used to assess the effect of PXN‐AS1 knock‐down on nuclear translocation of β‐catenin. Data in (D) were the quantification of bands in (C). E, The activity of Wnt/β‐catenin pathway in PXN‐AS1 silenced cells was estimated via TOP/FOP flash assay. F‐G, DKK1 mRNA and protein levels were tested by qRT‐PCR and Western blot after PXN‐AS1 silence or PXN‐AS1 overexpression. H, Expression of DKK1 in tumours from in vivo assays was detected via qRT‐PCR and Western blot analyses. I‐J, DKK1 expression was detected in cells transfected with sh‐SOX9 and pcDNA3.1/SOX9 via qRT‐PCR. **P < .01

SOX9 was reported to activate Wnt/β‐catenin pathway and drive the tumour progression in non‐small‐cell lung cancer.^19^ Hence, we explored the interaction between SOX9 and Wnt/β‐catenin pathway in GBM. As shown in Figure S3A, two well‐known downstream targets of Wnt/β‐catenin pathway, including cyclin D1 and c‐myc, demonstrated as remarkably decreased whereas CTNNB1 level unchanged in SOX9 down‐regulated cells. Besides, subcellular fractionation assay plus Western blot revealed that SOX9 knock‐down restrained the translocation of β‐catenin into nuclear (Figure S3B). And the suppressed nuclear translocation of β‐catenin under SOX9 silence was further confirmed via IF assay (Figure S3C). According to TOP/FOP flash assay, we found that the activity of Wnt/β‐catenin pathway was also repressed by sh‐SOX9 transfection (Figure S3D). Owing to the activated role of SOX9 in Wnt/β‐catenin pathway, we speculated that PXN‐AS1 exerted the same function on Wnt/β‐catenin pathway. Consistently, PXN‐AS1 silence hampered the mRNA expression of cyclin D1 and c‐myc but not that of CTNNB1, whereas restrained the protein levels of all the three genes (Figure 4A‐B). From subcellular fractionation plus Western blot analysis, nuclear translocation of β‐catenin was alleviated in cells with the transfection of sh‐PXN‐AS1 (Figure 4C‐D), so were the results in IF staining (Figure S3E). TOP/FOP flash assay further confirmed the repressive effect of PXN‐AS1 deficiency on Wnt/β‐catenin pathway (Figure 4E).

Thereafter, we aimed to figure out the downstream target of PXN‐AS1 in regulating Wnt signalling. Hence, a great number of genes regarding this pathway were evaluated in U87 and U251 cells under PXN‐AS1 inhibition. As a result, silencing PXN‐AS1 had no apparent influence on the level of various Wnt ligands (Figure S4A). Also, the expression of several genes which regulated β‐catenin level (such as FZD1, LRP5/6 and GSK3B) were not affected by PXN‐AS1 suppression; besides, several well‐known inhibitors of this pathway (including DKK3, SFPR1/2/4/5, WIF‐1 and HOPR1) kept nearly unchanged except that DDK1 expression was greatly enhanced by PXN‐AS1 depletion (Figure S4B). Moreover, DKK1 was widely known as an inhibitor gene of Wnt/β‐catenin pathway,^20^ and its mRNA and protein levels were facilitated with the transfection of sh‐PXN‐AS1 and diminished by PXN‐AS1 up‐regulation (Figure 4F‐G). Moreover, DKK1 expression in tumours with PXN‐AS1 silence was markedly increased (Figure 4H). Furthermore, inhibition of DKK1 countervailed the suppression of Wnt/β‐catenin pathway in PXN‐AS1‐silenced GBM cells (Figure S4C‐D), further validating that PXN‐AS1 regulated Wnt/β‐catenin in GBM through targeting DKK1. Meanwhile, SOX9 knock‐down or overexpression delineated the same effect on DKK1 mRNA and protein levels (Figure 4I‐J). Conclusively, SOX9‐enhanced PXN‐AS1 activates Wnt/β‐catenin pathway via DKK1‐dependent manner.

### 3.5 PXN‐AS1 recruits EZH2 to epigenetically suppress DKK1 expression

**FIGURE 5:**
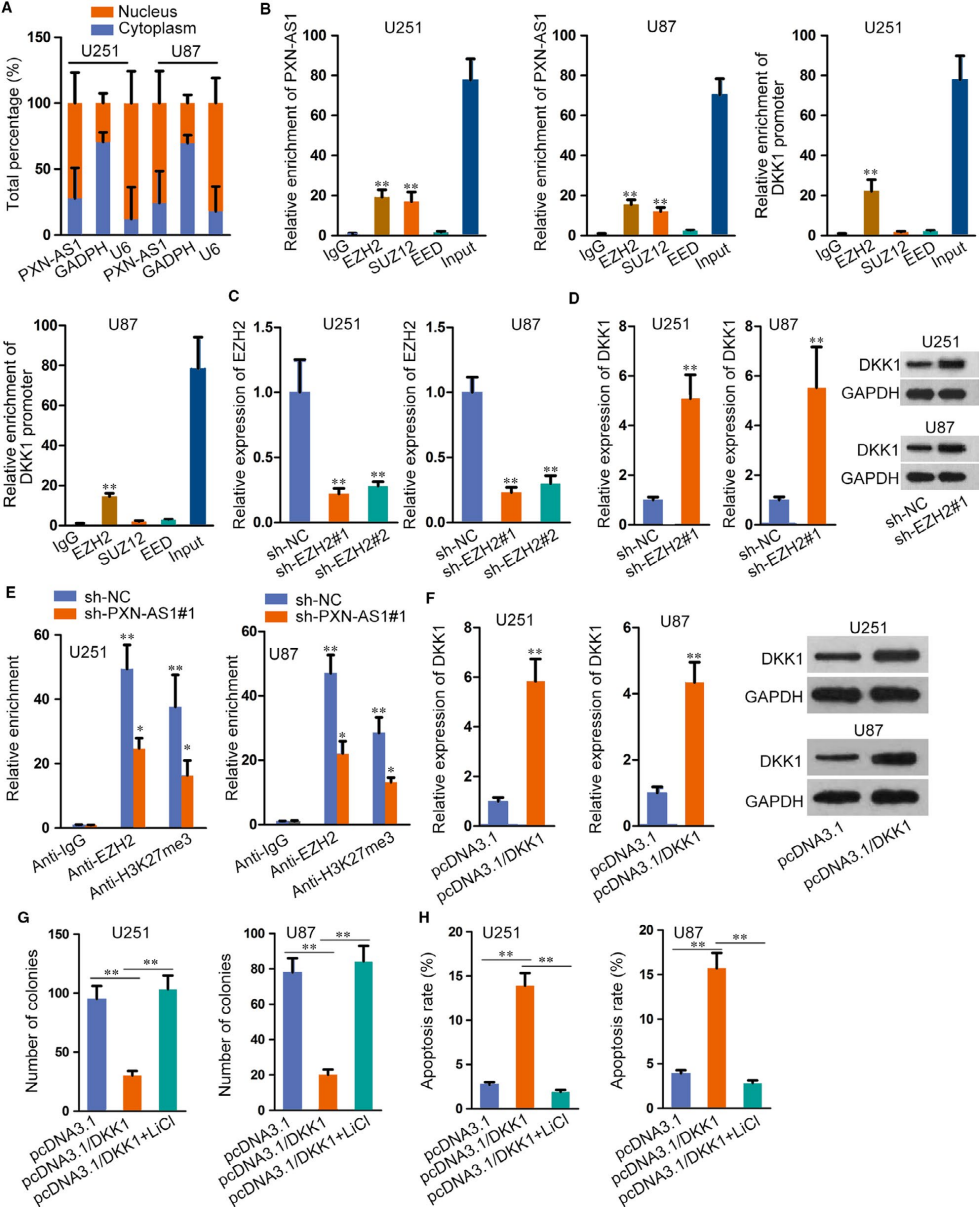
PXN‐AS1 recruits EZH2 to epigenetically suppress DKK1 expression. A, The distribution of PXN‐AS1 in GBM cells was determined subcellular fractionation assay. B, The enrichment of PXN‐AS1 or DKK1 promoter by EZH2/SUZ12/EED was determined by the use of RIP assay or ChIP assay, respectively. C, EZH2 knock‐down efficiency was evaluated by qRT‐PCR in GBM cells. D, DKK1 mRNA and protein levels were tested through qRT‐PCR and Western blot upon EZH2 depletion. (E) ChIP assay revealed the interaction between EZH2 and DKK1 promoter, and the interaction and H3K27me3 enrichment were tested in sh‐PXN‐AS1 and sh‐NC transfected cells. F, DKK1 expression was examined by qRT‐PCR and Western blot after transfecting overexpression plasmids. G, Cell proliferation was measured via colony formation assay in cells treated indicated plasmids. H, Cell apoptosis was assessed through flow cytometry analysis in cells after transfecting indicated plasmids. *P < .05, **P < .01

On basis of regulatory role of PXN‐AS1 in DKK1 expression, we investigated the underlying regulatory mechanism of PXN‐AS1 on DKK1. Firstly, we detected the distribution of PXN‐AS1 in GBM cells. As a result, PXN‐AS1 was mainly localized in the nucleus of U251 and U87 cells (Figure 5A), which implied that PXN‐AS1 could participate in transcriptional regulation. Abundant literatures revealed that lncRNAs exerted regulatory functions via binding to histone modification enzymes. Recent evidence has showed that lncRNAs could modulate downstream genes via interacting with PRC2, which is a methyltransferase that represses the transcription of specific genes by trimethylating H3K27. Therefore, we carried out RIP assay to probe whether PXN‐AS1 interacted with PRC2. Three key components of PRC2 (EZH2, SUZ12 and EED) were used in the study. The results delineated that both PXN‐AS1 and DKK1 promoter could bind to EZH2 in U251 and U87 cells (Figure 5B). Besides, we transfected sh‐EZH2 into GBM cells for down‐regulating its expression (Figure 5C). Further, we found that DKK1 mRNA and protein levels were enhanced in sh‐EZH2 transfected cells (Figure 5D). From ChIP assay, it was showed that EZH2 directly combined with DKK1 promoter region and histone H3 lysine 27 trimethylation (H3K27me3) was also abundant at DKK1 promoter in U251 and U87 cells. Meanwhile, PXN‐AS1 knock‐down reduced EZH2 occupancy and H3K27me3 enrichment on DKK1 promoter region (Figure 5E). Later, we overexpressed DKK1 expression (Figure 5F) and used Wnt/β‐catenin pathway activator (LiCl) to conduct further study. As illustrated in Figure 5G, the treatment of LiCl countered DKK1 overexpression‐mediated suppression in GBM cell proliferation. Furthermore, induced apoptosis in DKK1 up‐regulated cells was reserved by treating LiCl (Figure 5H). Collectively, PXN‐AS1 recruited EZH2 to epigenetically suppress DKK1 expression.

### 3.6 PXN‐AS1 promotes the GBM progression by epigenetically silencing DKK1

**FIGURE 6:**
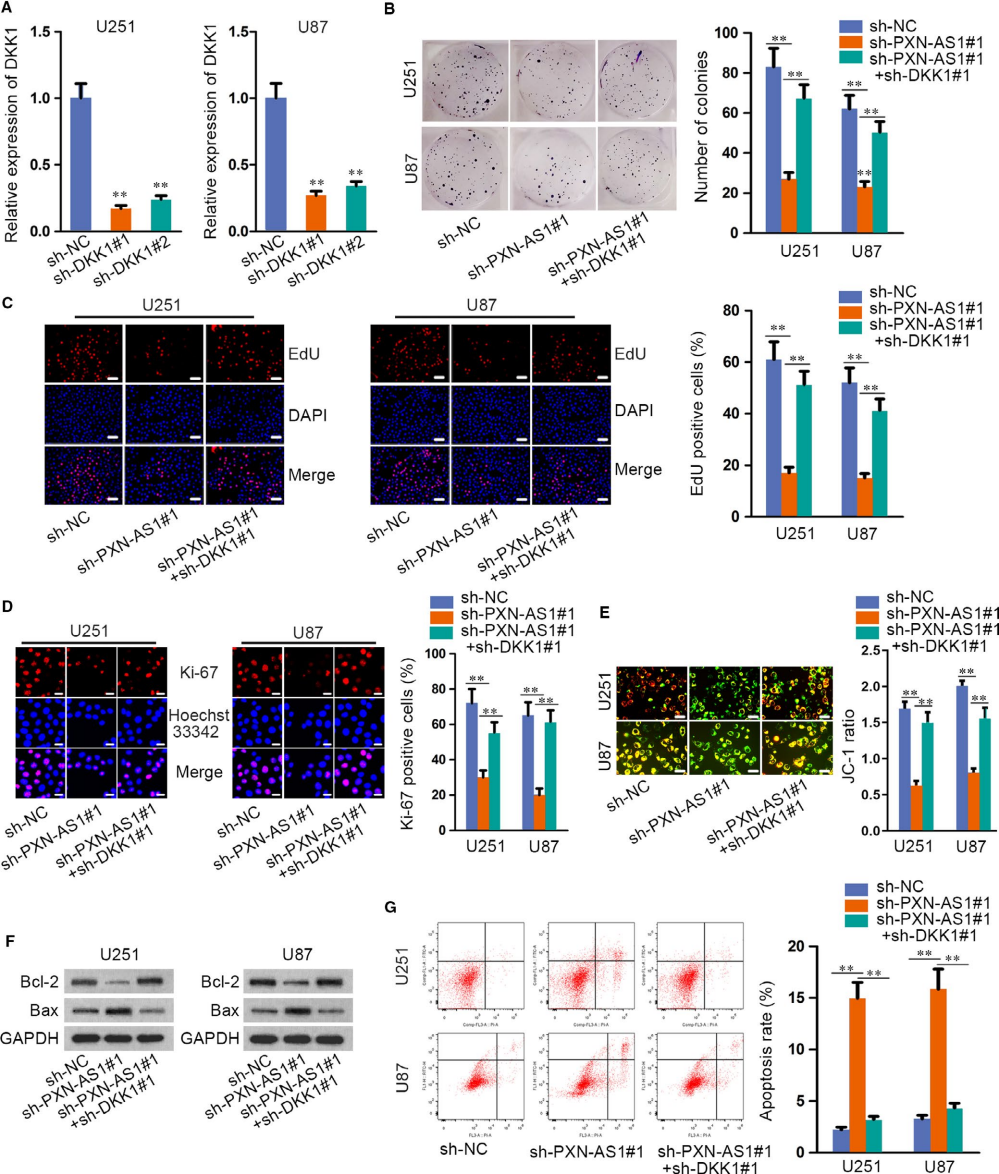
PXN‐AS1 promotes GBM progression by epigenetically silencing DKK1. A, Transfection efficiency of sh‐DKK1 was tested. B‐D, Cell proliferation in cells transfected with sh‐NC, sh‐PXN‐AS1, sh‐PXN‐AS1 + pcDNA3.1/DKK1 was tested by colony formation assay, EdU assay and immunofluorescence. Scale bar = 100μm. E‐G, JC‐1, Western blot and flow cytometry assays were utilized to determine cell apoptosis in cells transfected with sh‐NC, sh‐PXN‐AS1, sh‐PXN‐AS1 + pcDNA3.1/DKK1. Scale bar = 100μm. **P < .01

Finally, restoration experiments were designed and carried out to verify that PXN‐AS1 regulated the proliferation and apoptosis of GBM via epigenetically suppressing DKK1 expression. Firstly, the expression of DKK1 was stably silenced in U251 and U87 cells by transfecting sh‐DKK1 (Figure 6A). Through colony formation assay, EdU assay and immunofluorescence, the results manifested that down‐regulated DKK1 rescued the repressive proliferation of sh‐PXN‐AS1 transfected cells (Figure 6B‐D). Additionally, JC‐1, Western blot and flow cytometry assays unveiled that the promoted effect of PXN‐AS1 depletion on cell apoptosis was countervailed by DKK1 silencing (Figure 6E‐G). All data disclosed that PXN‐AS1 promoted GBM progression by epigenetically silencing DKK1.

## 4 DISCUSSION

In recent years, extensive reports have elucidated that lncRNAs undergo the main responsibility for triggering the dysregulation of various cancers. For example, lncRNA CCAT2 was reported to boost the proliferation and migration via regulating TGF‐β signalling pathway in breast cancer.^10^ LncRNA AK023391 activated PI3K/Akt signalling pathway in gastric cancer and promoted cancer progression.^21^ LncRNA UICLM facilitated liver metastasis in colorectal cancer by targeting miRNA‐215/ZEB2 axis.^22^ Nonetheless, the explicit role and molecular mechanism of lncRNA PXN‐AS1 underlying GBM progression have not been explored. The current study manifested that PXN‐AS1 was highly overexpressed in GBM cells, and down‐regulated PXN‐AS1 hampered cell proliferation and enhanced cell apoptosis in GBM. Furthermore, PXN‐AS1 promoted GBM tumour growth in vivo. More importantly, the expression of PXN‐AS1 was confirmed to be transcriptionally activated by SOX9. In brief, SOX9‐activated PXN‐AS1 exhibited the carcinogenic property in GBM. Interestingly, the function of PXN‐AS1 changes in different cancer types, which might be attributed to tumour heterogeneity to some extent. In detail, PXN‐AS1 played a promoting role in the tumorigenesis of lung cancer^23^ and nasopharyngeal carcinoma,^24^ similar to the findings observed in current study. However, a recent report also suggested that PXN‐AS1 served as a tumour suppressor in pancreatic cancer.^15^

As an important biological participator, Wnt/β‐catenin pathway exerts huge functions in the process of tumour initiation and progression.^25^, ^26^, ^27^ Among which, its functional contribution to lncRNAs‐mediated cancer development has attracted people's attention.^28^ Through researches of numerous years, it was confirmed that enhanced nuclear translocation of β‐catenin indicates the activation of Wnt/β‐catenin pathway.^29^ The transcription factor SOX9 has been reported to activate Wnt/β‐catenin pathway in non‐small‐cell lung cancer.^19^ In this regard, we investigated whether SOX9 and SOX9‐mediated PXN‐AS1 could act the similar effect on GBM progression. The results showed that SOX9 knock‐down effectively inhibited the expression of relative genes in Wnt pathway, and the nuclear translocation of β‐catenin was also restrained by down‐regulated SOX9. Consistently, the same results were also observed in PXN‐AS1 silenced GBM cells. As an inhibitor gene of Wnt/β‐catenin pathway, the expression of DKK1 was significantly reduced by the overexpression of SOX9 and PXN‐AS1. Taken together, PXN‐AS1 could activate Wnt/β‐catenin pathway in GBM via prompting β‐catenin to translocate into nucleus.

Localized in nuclear, PXN‐AS1 was seemed to regulate gene expression in transcriptional level. A growing number of evidence suggested that lncRNAs could interact with various histone modification enzymes to impact cellular functions and regulate target gene expression.^30^, ^31^, ^32^ PRC2, containing EZH2, SUZ12 and EED, is a classical methyltransferase for histone H3K27me3.^33^ For instance, LINC00470 distracts cell autophagy in GBM by epigenetically regulating ELFN2.^34^ LncRNA HOTAIR promotes epithelial‐to‐mesenchymal transition and regulates H3K27me3 in gastric cancer.^35^ LncRNA CASC15 epigenetically regulates PDCD4 expression by recruiting EZH2 and promotes melanoma progression.^36^ In this study, we found that PXN‐AS1 interacted with EZH2, and EZH2 silence decreased DKK1 expression. Importantly, PXN‐AS1 knock‐down reduced EZH2 interaction and the enrichment of H3K27me3 in DKK1 promoter region. Further, it was discovered that the treatment of LiCl rescued the promoting effect of DKK1 overexpression on GBM cell growth. As observed in restoration assays, up‐regulated DKK1 counteracted PXN‐AS1 silence‐mediated suppression on GBM cell growth.

Conclusively, PXN‐AS1 acts as an oncogene in GBM, and SOX9‐activated PXN‐AS1 promotes GBM progression by epigenetically silencing DKK1, suggesting a helpful revelation for the exploration of novel GBM diagnostic and therapeutic strategies.

## CONFLICT OF INTEREST

The authors confirm that there are no conflicts of interest.

## AUTHORS’ CONTRIBUTION

Hongjin Chen and Guoqiang Hou wrote the paper; Jian Yang, Weilin Chen and Liemei Guo performed the research; Qin Mao designed the research study; Jianwei Ge and Xiaohua Zhang contributed essential reagents and analysed the data.

## Supporting information

Figure S1

Figure S2

Figure S3

Figure S4

Table S1

## Data Availability

Research data are not shared.

## References

[1] Abou‐Antoun TJ , Hale JS , Lathia JD , Dombrowski SM . Brain Cancer stem cells in adults and children: cell biology and therapeutic implications. Neurotherapeutics. 2017;14:372‐384. 28374184 10.1007/s13311-017-0524-0 PMC5398995

[2] Shergalis A , Bankhead A III , Luesakul U , Muangsin N , Neamati N . Current challenges and opportunities in treating glioblastoma. Pharmacol Rev. 2018;70:412‐445. 29669750 10.1124/pr.117.014944 PMC5907910

[3] Liu X , Zheng J , Xue Y , et al. Inhibition of TDP43‐Mediated SNHG12‐miR‐195‐SOX5 feedback loop impeded malignant biological behaviors of glioma cells. Mol Ther Nucl Acids. 2018;10:142‐158. 10.1016/j.omtn.2017.12.001 PMC5751968 29499929

[4] Gao X , Guo X , Xue H , et al. lncTCF7 is a negative prognostic factor, and knockdown of lncTCF7 inhibits migration, proliferation and tumorigenicity in glioma. Sci Rep. 2017;7:17456. 29234033 10.1038/s41598-017-17340-y PMC5727168

[5] Bartonicek N , Maag JL , Dinger ME . Long noncoding RNAs in cancer: mechanisms of action and technological advancements. Mol Cancer. 2016;15:43. 27233618 10.1186/s12943-016-0530-6 PMC4884374

[6] Adams BD , Parsons C , Walker L , Zhang WC , Slack FJ . Targeting noncoding RNAs in disease. J Clin Investig. 2017;127:761‐771. 28248199 10.1172/JCI84424 PMC5330746

[7] Ning L , Li Z , Wei D , Chen H , Yang C . LncRNA, NEAT1 is a prognosis biomarker and regulates cancer progression via epithelial‐mesenchymal transition in clear cell renal cell carcinoma. Cancer Biomark. 2017;19:75‐83. 28269753 10.3233/CBM-160376 PMC13020697

[8] Zhang S , Wang Y , Chen M , et al. CXCL12 methylation‐mediated epigenetic regulation of gene expression in papillary thyroid carcinoma. Sci Rep. 2017;7:44033. 28272462 10.1038/srep44033 PMC5356381

[9] Xiao JN , Yan TH , Yu RM , et al. Long non‐coding RNA UCA1 regulates the expression of Snail2 by miR‐203 to promote hepatocellular carcinoma progression. J Cancer Res Clin Oncol. 2017;143:981‐990. 28271214 10.1007/s00432-017-2370-1 PMC11818964

[10] Wu ZJ , Li Y , Wu YZ , et al. Long non‐coding RNA CCAT2 promotes the breast cancer growth and metastasis by regulating TGF‐beta signaling pathway. Eur Rev Med Pharmacol Sci. 2017;21:706‐714. 28272713

[11] Fu M , Huang Z , Zang X , et al. Long noncoding RNA LINC00978 promotes cancer growth and acts as a diagnostic biomarker in gastric cancer. Cell Prolif. 2018;51. 10.1111/cpr.12425 PMC6528885 29271006

[12] Li J , Lian Y , Yan C , et al. Long non‐coding RNA FOXP4‐AS1 is an unfavourable prognostic factor and regulates proliferation and apoptosis in colorectal cancer. Cell Prolif. 2017;50(1):e12312. 10.1111/cpr.12312 PMC6529074 27790757

[13] Liu C , Zhang Y , She X , et al. A cytoplasmic long noncoding RNA LINC00470 as a new AKT activator to mediate glioblastoma cell autophagy. J Hematol Oncol. 2018;11:77. 29866190 10.1186/s13045-018-0619-z PMC5987392

[14] Li Q , Dong C , Cui J , Wang Y , Hong X . Over‐expressed lncRNA HOTAIRM1 promotes tumor growth and invasion through up‐regulating HOXA1 and sequestering G9a/EZH2/Dnmts away from the HOXA1 gene in glioblastoma multiforme. J Exp Clin Cancer Res. 2018;37:265. 30376874 10.1186/s13046-018-0941-x PMC6208043

[15] Yan J , Jia Y , Chen H , Chen W , Zhou X . Long non‐coding RNA PXN‐AS1 suppresses pancreatic cancer progression by acting as a competing endogenous RNA of miR‐3064 to upregulate PIP4K2B expression. J Exp Clin Cancer Res. 2019;38:390. 31488171 10.1186/s13046-019-1379-5 PMC6727519

[16] Lakhani SA , Masud A , Kuida K , et al. Caspases 3 and 7: key mediators of mitochondrial events of apoptosis. Science. 2006;311:847‐851. 16469926 10.1126/science.1115035 PMC3738210

[17] Hiraoka K , Hayashi T , Kaneko R , et al. SOX9‐mediated upregulation of LGR5 is important for glioblastoma tumorigenicity. Biochem Biophys Res Comm. 2015;460:216‐221. 25770425 10.1016/j.bbrc.2015.03.012

[18] Wang J , Xu SL , Duan JJ , et al. Invasion of white matter tracts by glioma stem cells is regulated by a NOTCH1‐SOX2 positive‐feedback loop. Nat Neurosci. 2019;22:91‐105. 30559479 10.1038/s41593-018-0285-z

[19] Huang JQ , Wei FK , Xu XL , et al. SOX9 drives the epithelial‐mesenchymal transition in non‐small‐cell lung cancer through the Wnt/beta‐catenin pathway. J Transl Med. 2019;17:143. 31060551 10.1186/s12967-019-1895-2 PMC6501400

[20] Elliott C , Rojo AI , Ribe E , et al. A role for APP in Wnt signalling links synapse loss with beta‐amyloid production. Transl Psychiat. 2018;8:179. 10.1038/s41398-018-0231-6 PMC6145937 30232325

[21] Huang Y , Zhang J , Hou L , et al. LncRNA AK023391 promotes tumorigenesis and invasion of gastric cancer through activation of the PI3K/Akt signaling pathway. J Exp Clin Cancer Res. 2017;36:194. 29282102 10.1186/s13046-017-0666-2 PMC5745957

[22] Chen DL , Lu YX , Zhang JX , et al. Long non‐coding RNA UICLM promotes colorectal cancer liver metastasis by acting as a ceRNA for microRNA‐215 to regulate ZEB2 expression. Theranostics. 2017;7:4836‐4849. 29187907 10.7150/thno.20942 PMC5706103

[23] Zhang Z , Peng Z , Cao J , et al. Long noncoding RNA PXN‐AS1‐L promotes non‐small cell lung cancer progression via regulating PXN. Cancer Cell Int. 2019;19:20‐. 30679933 10.1186/s12935-019-0734-0 PMC6341638

[24] Jia X , Niu P , Xie C , Liu H . Long noncoding RNA PXN‐AS1‐L promotes the malignancy of nasopharyngeal carcinoma cells via upregulation of SAPCD2. Cancer Med. 2019;8:4278‐4291. 31173488 10.1002/cam4.2227 PMC6675719

[25] Chen Q , Cai J , Wang Q , et al. Long noncoding RNA NEAT1, regulated by the EGFR pathway, contributes to glioblastoma progression through the WNT/beta‐catenin pathway by scaffolding EZH2. Clin Cancer Res. 2018;24:684‐695. 29138341 10.1158/1078-0432.CCR-17-0605

[26] Yan X , Zhang D , Wu W , et al. Mesenchymal stem cells promote Hepatocarcinogenesis via lncRNA‐MUF interaction with ANXA2 and miR‐34a. Can Res. 2017;77:6704‐6716. 10.1158/0008-5472.CAN-17-1915 28947421

[27] Malakar P , Shilo A , Mogilevsky A , et al. Long noncoding RNA MALAT1 promotes hepatocellular carcinoma development by SRSF1 upregulation and mTOR activation. Can Res. 2017;77:1155‐1167. 10.1158/0008-5472.CAN-16-1508 PMC5334181 27993818

[28] Zhang Z , Zhou C , Chang Y , et al. Long non‐coding RNA CASC11 interacts with hnRNP‐K and activates the WNT/beta‐catenin pathway to promote growth and metastasis in colorectal cancer. Cancer Lett. 2016;376:62‐73. 27012187 10.1016/j.canlet.2016.03.022

[29] Arima M , Hasegawa D , Yoshida S , et al. R‐spondin 2 promotes osteoblastic differentiation of immature human periodontal ligament cells through the Wnt/beta‐catenin signaling pathway. J Periodontal Res. 2019;54:143‐153. 30284717 10.1111/jre.12611

[30] Sun M , Nie F , Wang Y , et al. LncRNA HOXA11‐AS Promotes Proliferation and Invasion of Gastric Cancer by Scaffolding the Chromatin Modification Factors PRC2, LSD1, and DNMT1. Can Res. 2016;76:6299‐6310. 10.1158/0008-5472.CAN-16-0356 27651312

[31] Shen Y , Wang Z , Loo LW , et al. LINC00472 expression is regulated by promoter methylation and associated with disease‐free survival in patients with grade 2 breast cancer. Breast Cancer Res Treat. 2015;154:473‐482. 26564482 10.1007/s10549-015-3632-8 PMC4854534

[32] Khalil AM , Guttman M , Huarte M , et al. Many human large intergenic noncoding RNAs associate with chromatin‐modifying complexes and affect gene expression. Proc Natl Acad Sci USA. 2009;106:11667‐11672. 19571010 10.1073/pnas.0904715106 PMC2704857

[33] Conway E , Healy E , Bracken AP . PRC2 mediated H3K27 methylations in cellular identity and cancer. Curr Opin Cell Biol. 2015;37:42‐48. 26497635 10.1016/j.ceb.2015.10.003

[34] Liu C , Fu H , Liu X , et al. LINC00470 Coordinates the Epigenetic Regulation of ELFN2 to Distract GBM Cell Autophagy. Mol Ther. 2018;26:2267‐2281. 30037656 10.1016/j.ymthe.2018.06.019 PMC6127511

[35] Song Y , Wang R , Li LW , et al. Long non‐coding RNA HOTAIR mediates the switching of histone H3 lysine 27 acetylation to methylation to promote epithelial‐to‐mesenchymal transition in gastric cancer. Int J Oncol. 2019;54:77‐86. 30431069 10.3892/ijo.2018.4625 PMC6254860

[36] Yin Y , Zhao B , Li D , Yin G . Long non‐coding RNA CASC15 promotes melanoma progression by epigenetically regulating PDCD4. Cell Biosci. 2018;8:42. 30013768 10.1186/s13578-018-0240-4 PMC6044067

