# Supplementary figures and images for: SOX9‐activated PXN‐AS1 promotes the tumorigenesis of glioblastoma by EZH2‐mediated methylation of DKK1
Source: J Cell Mol Med. 2020 Apr 23;24(11):6070–82. doi: 10.1111/jcmm.15189 (PMC7294137; doi:10.1111/jcmm.15189)

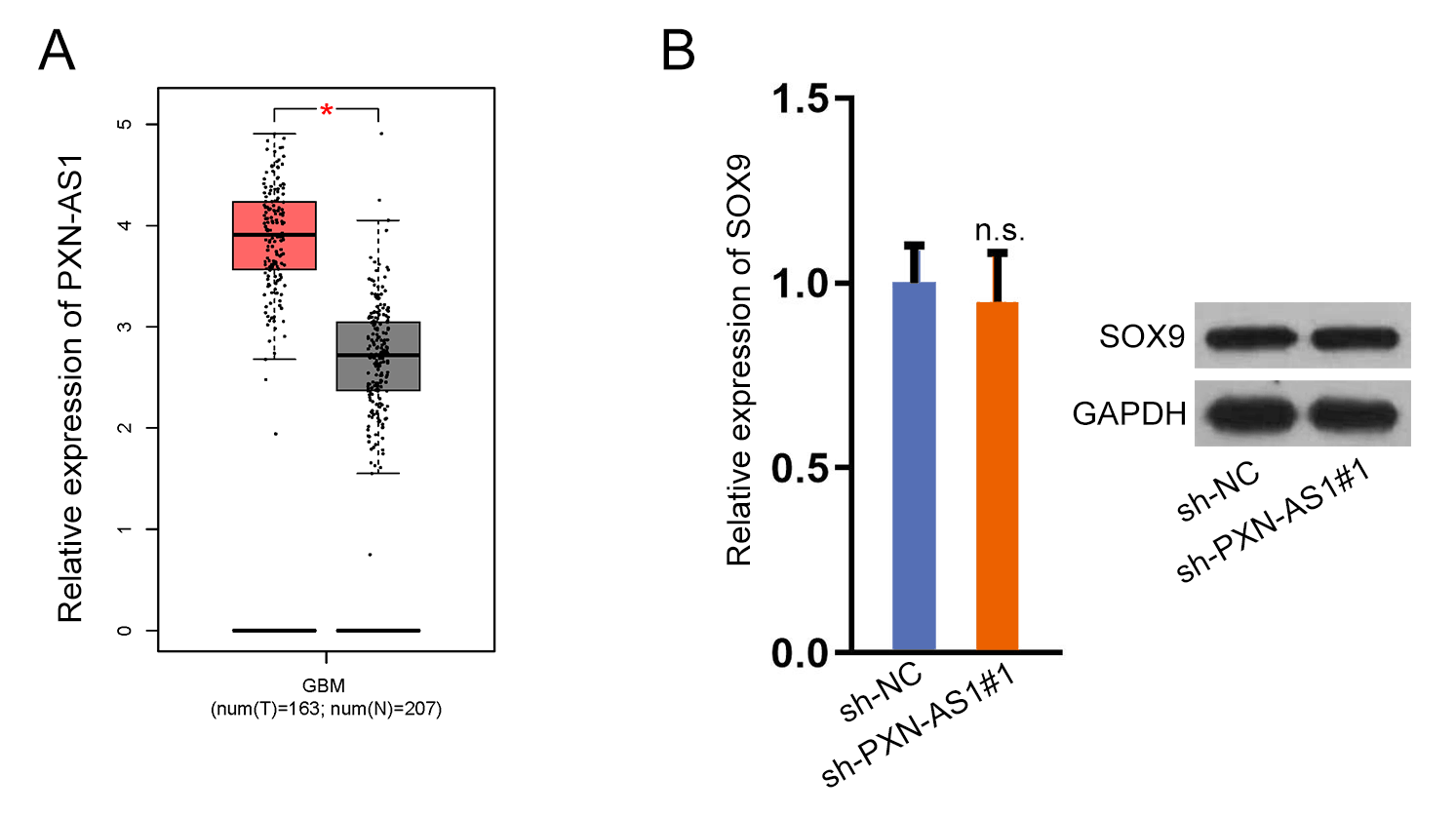

Supplement: Supplementary file 1 — Figure S1 [file JCMM-24-6070-s001.tif]

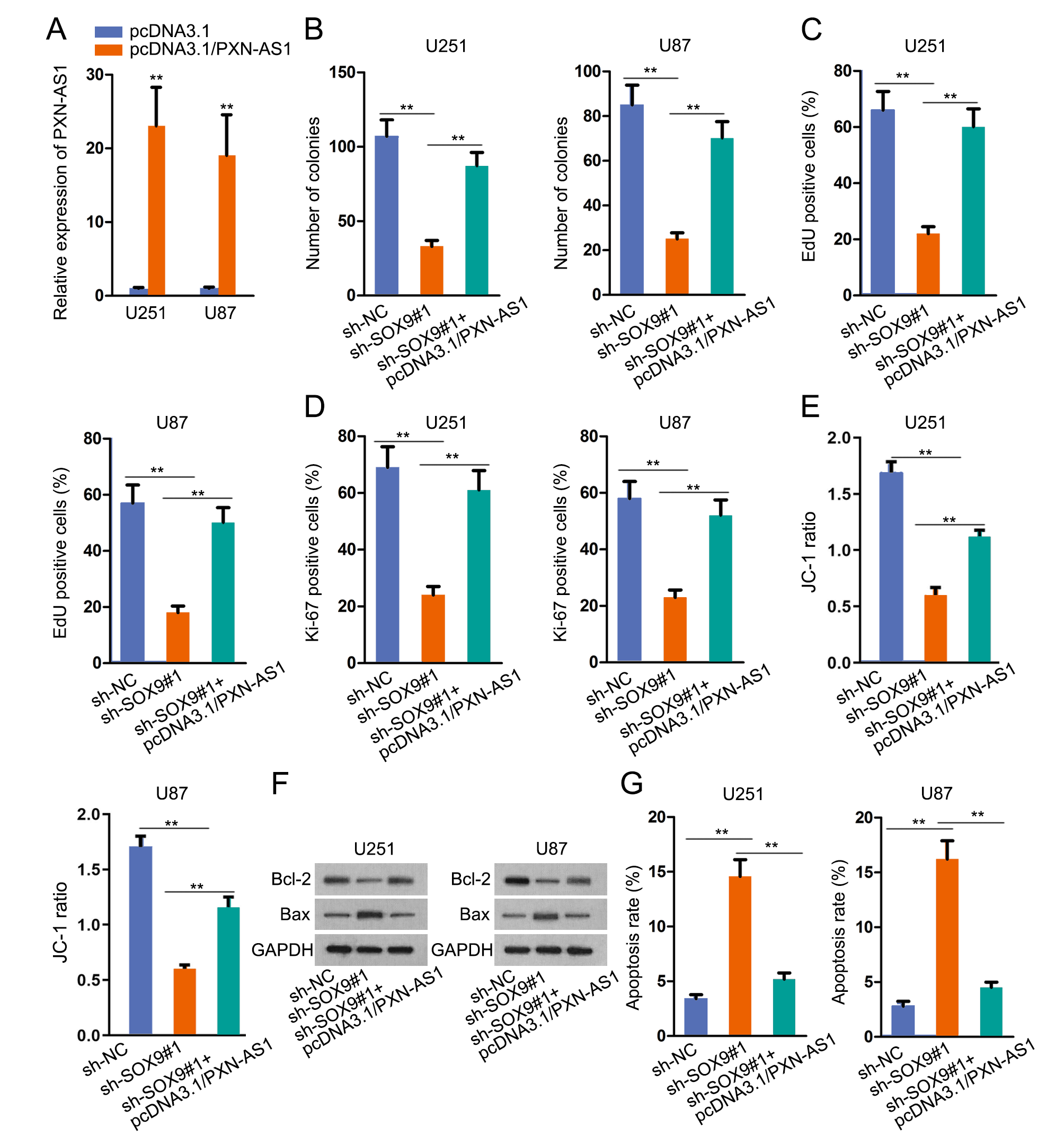

Supplement: Supplementary file 2 — Figure S2 [file JCMM-24-6070-s002.tif]

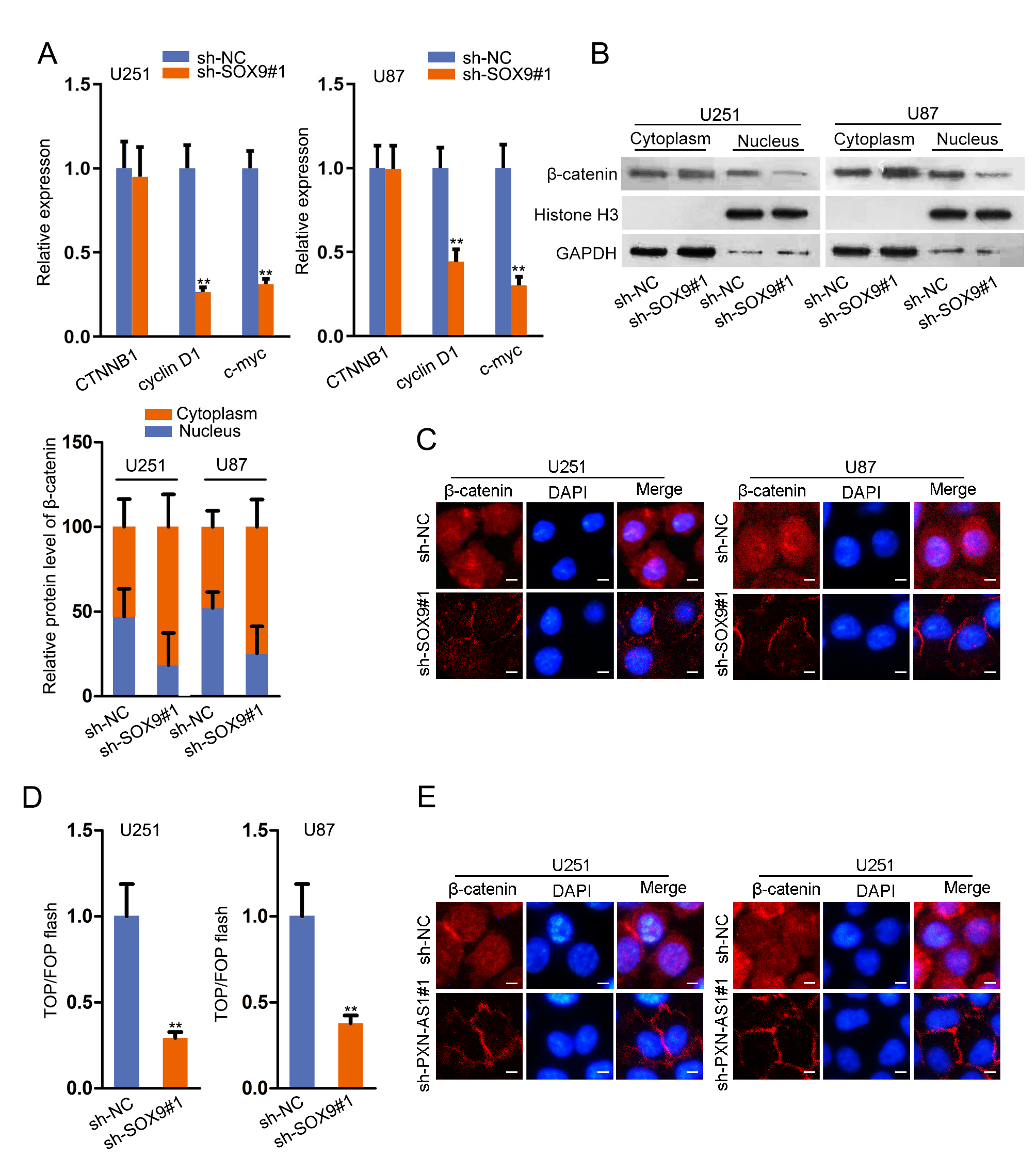

Supplement: Supplementary file 3 — Figure S3 [file JCMM-24-6070-s003.tif]

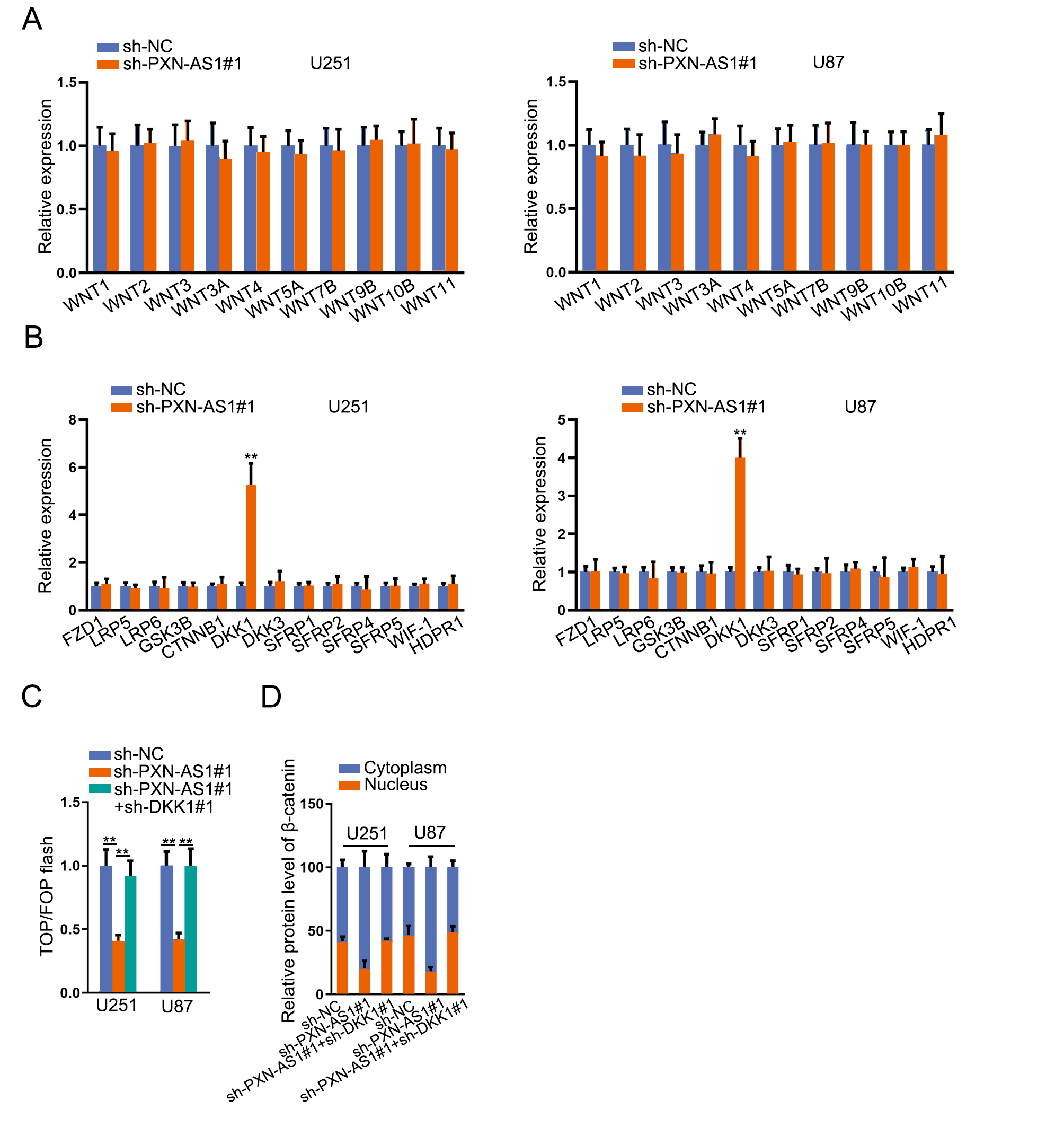

Supplement: Supplementary file 4 — Figure S4 [file JCMM-24-6070-s004.tif]
