# Supplementary material for: SOX9‐activated PXN‐AS1 promotes the tumorigenesis of glioblastoma by EZH2‐mediated methylation of DKK1
Source: J Cell Mol Med. 2020 Apr 23;24(11):6070–82. doi: 10.1111/jcmm.15189 (PMC7294137; doi:10.1111/jcmm.15189)
Supplement: Supplementary file 5 — Table S1 [file JCMM-24-6070-s005.docx]

**Sequences of primers used in qRT-PCR**

| **Gene** | **Forward primer (5'-3')** | **Reverse primer (5'-3')** |
| --- | --- | --- |
| PXN-AS1 | AGAAGCTGCCGTTCACGAAT | ATGGGAAACGCCTAGCAAGTA |
| SOX9 | AGGAAGCTCGCGGACCAGTAC | GGTGGTCCTTCTTGTGCTGCAC |
| CTNNB1 | CACAAGCAGAGTGCTGAAGGTG | GATTCCTGAGAGTCCAAAGACAG |
| CCND1 | TCTACACCGACAACTCCATCCG | TCTGGCATTTTGGAGAGGAAGTG |
| c-Myc | CCTGGTGCTCCATGAGGAGAC | CAGACTCTGACCTTTTGCCAGG |
| DKK1 | GGTATTCCAGAAGAACCACCTTG | CTTGGACCAGAAGTGTCTAGCAC |
| EZH2 | GACCTCTGTCTTACTTGTGGAGC | CGTCAGATGGTGCCAGCAATAG |
| WNT1 | CTCTTCGGCAAGATCGTCAACC | CGATGGAACCTTCTGAGCAGGA |
| WNT2 | AGGATGCCAGAGCCCTGATGAA | AGCCAGCATGTCCTGAGAGTAC |
| WNT3 | GCGTGTTAGTGTCCAGGGAGTT | TGAGGTGCATGTGGTCCAGGAT |
| WNT3A | ATGAACCGCCACAACAACGAGG | GTCCTTGAGGAAGTCACCGATG |
| WNT4 | GCTGGAGAAGTGCGGCTGTGA | CCACAAACGACTGTGAGAAGGC |
| WNT5A | TACGAGAGTGCTCGCATCCTCA | TGTCTTCAGGCTACATGAGCCG |
| WNT7B | AGAAGACCGTCTTCGGGCAAGA | AGTTGCTCAGGTTCCCTTGGCT |
| WNT9B | CCTGCTTGAGTGCCAGTTTCAG | ACACCGCGTACAGGAAAGCTGT |
| WNT10B | CTCGGGATTTCTTGGATTCCAGG | GCCATGACACTTGCATTTCCGC |
| WNT11 | CTGTGAAGGACTCGGAACTCGT | AGCTGTCGCTTCCGTTGGATGT |
| FZD1 | GCTTTGTGTCGCTCTTCCGCAT | TACAGCACGCTGAAGACGCCAA |
| LRP5 | GGACACCAACATGATCGAGTCG | CGCTCAATGCTGTGCAGATTCC |
| LRP6 | CAGTTGGAGTGGTGCTGAAAGG | CCATCCAAAGCAGCCCGTTCAA |
| GSK3B | CCGACTAACACCACTGGAAGCT | AGGATGGTAGCCAGAGGTGGAT |
| DKK3 | GTGCATCATCGACGAGGACTGT | TGGTCTCCACAGCACTCACTGT |
| SFRP1 | CAATGCCACCGAAGCCTCCAAG | CAAACTCGCTGGCACAGAGATG |
| SFRP2 | CTCCAAAGGTATGTGAAGCCTGC | CCAGGATGATTTTGGTATCTCGG |
| SFRP4 | CTATGACCGTGGCGTGTGCATT | GCTTAGGCGTTTACAGTCAACATC |
| SFRP5 | CTGAGATGCTGCACTGCCACAA | GTCAGCACTGTGCTCCATCTCA |
| WIF-1 | GGTGCCGAAATGGAGGCTTTTG | GATGCAGAAACCAGGAGTCACAC |
| HDPR1 | CTGAAGAGCACCTGGAGACAGA | GCAAAAGCAGGTGCTGGAATGAC |
